# Colonization Dynamics of Multidrug-Resistant *Klebsiella pneumoniae* Are Dictated by Microbiota-Cluster Group Behavior over Individual Antibiotic Susceptibility: A Metataxonomic Analysis

**DOI:** 10.3390/antibiotics10030268

**Published:** 2021-03-07

**Authors:** János Juhász, Balázs Ligeti, Márió Gajdács, Nóra Makra, Eszter Ostorházi, Ferenc Balázs Farkas, Balázs Stercz, Ákos Tóth, Judit Domokos, Sándor Pongor, Dóra Szabó

**Affiliations:** 1 Institute of Medical Microbiology, Faculty of Medicine, Semmelweis University, Nagyvárad tér 4., 1089 Budapest, Hungary; juhaszjanos4@gmail.com (J.J.); obalasz@gmail.com (B.L.); gajdacs.mario@szte.hu (M.G.); nora.makra@indamail.hu (N.M.); droeszter@gmail.com (E.O.); balazsqb@gmail.com (F.B.F.); stercz.balazs@med.semmelweis-univ.hu (B.S.); djudit90@gmail.com (J.D.); 2 Faculty of Information Technology and Bionics, Pázmány Péter Catholic University, Práter utca 50/A, 1083 Budapest, Hungary; pongor.sandor@itk.ppke.hu; 3 Department of Pharmacodynamics and Biopharmacy, Faculty of Pharmacy, University of Szeged, Eötvös utca 6, 6720 Szeged, Hungary; 4 Department of Bacteriology, Mycology and Parasitology, National Public Health Centre, Albert Flórián út 2-6, 1097 Budapest, Hungary; toth.akos@nnk.gov.hu

**Keywords:** colonization, decolonization, gut microbiota, microbiota-cluster, antibiotic-induced microbiota alteration, metataxonomic analysis, MDR, Klebsiella pneumoniae

## Abstract

Gastrointestinal carriage of multidrug-resistant (MDR) bacteria is one of the main risk factors for developing serious, difficult-to-treat infections. Given that there is currently no all-round solution to eliminate colonization with MDR bacteria, it is particularly important to understand the dynamic process of colonization to aid the development of novel decolonization strategies. The aim of our present study was to perform metataxonomic analyses of gut microbiota dynamics during colonization with an extended-spectrum β-lactamase (ESBL)- and carbapenemase-producing *Klebsiella pneumoniae* (ECKP) strain in mice; additionally, to ascertain the effects of antibiotic administration (ampicillin, ceftazidime, and ciprofloxacin) on the establishment and elimination of ECKP intestinal colonization. We have found that the phyla Bacteroidetes and Firmicutes were most dominant in all of the treatment groups; however, Bacteroidetes was more common in the groups treated with antibiotics compared to the control group. Significant differences were observed among the different antibiotic-treated groups in beta but not alpha diversity, implying that the difference is the relative abundance of some bacterial community members. Bacteria from the *Lachnospiraceae* family (including *Agathobacter*, *Anaerostipes*, *Lachnoclostridium* 11308, *Lachnospiraceae* UCG-004, *Lachnospiraceae* NK3A20 group 11318, *Lachnospiraceae* NK4A136 group 11319, *Roseburia,* and *Tyzzerella*) showed an inverse relationship with the carriage rate of the ECKP strain, whereas members of Enterobacteriaceae and the ECKP strain have shown a correlational relationship. Our results suggest that the composition of the microbial community plays a primary role in the MDR-colonization rate, whereas the antibiotic susceptibility of individual MDR strains affects this process to a lesser extent. Distinct bacterial families have associated into microbial clusters, collecting taxonomically close species to produce survival benefits in the gut. These associations do not develop at random, as they may be attributed to the presence of specific metabolomic networks. A new concept should be introduced in designing future endeavors for MDR decolonization, supplemented by knowledge of the composition of the host bacterial community and the identification of bacterial clusters capable of suppressing or enhancing the invader species.

## 1. Introduction

The emergence and spread of multidrug-resistant (MDR) bacteria represent a serious public health issue worldwide. The production of extended-spectrum β-lactamase (ESBL; conferring resistance to most oxyimino-cephalosporins, e.g., ceftriaxone, cefotaxime, and ceftazidime) and carbapenemase (conferring resistance to almost all members of β-lactam antibiotics) enzymes in the members of Enterobacterales is one of the most challenging; the presence of these resistance determinants is most common in *Klebsiella* spp. and *Escherichia coli* [1,2,3]. These bacteria (i.e., ESBL-producers and carbapenemase-producing Enterobacterales (CRE)) present a considerable clinical problem, as the useful therapeutic armamentarium in these infections is very limited [4,5,6]. Many studies highlighted that infections caused by these pathogens are associated with high morbidity and mortality, and decreased quality of life in the affected patients, especially in vulnerable populations, such as pediatric patients, the elderly, the chronically ill, hospitalized patients, transplant recipients, and the immunosuppressed [7,8,9,10]. Unfortunately, resistance rates of these highly dangerous pathogens are on the rise both in hospital and community settings, particularly in Asian countries [2,11,12,13,14]. In fact, ESBL- and carbapenemase-producing *Klebsiella* has been designated as Priority 1: Critical on the World Health Organization (WHO) Global Priority List for antibiotic-resistant pathogens for the development of novel antibiotics [15].

The human gastrointestinal tract is a complex and dynamic ecological niche, colonized by numerous bacteria, which may significantly influence the physiology of its host and has many beneficial roles. One of these critical roles is the prevention of infections by exogenous pathogens through a mechanism called colonization resistance; members across the Firmicutes, Bacteroidetes, and Actinobacteria phyla have been identified to contribute to this mechanism of colonization resistance [16]. It has been suggested that these commensals may activate the body’s immune responses that will subsequently target pathogenic bacteria. They can produce inhibitory substances or directly compete with these pathogens for nutrients in the gut [17]. Nonetheless, the microbiota of the gut may also include opportunistic pathogens and may carry drug resistance genes [18]. Numerous recent publications have reported that healthy hosts may be asymptomatically colonized with ESBL-producers, with an overall colonization rate of 14%, which is more than a 5% increase from previous studies; there are important geographical differences in the prevalence. In Asia and Africa, the colonization rate may be as high as 46%, whereas in Europe and the Americas, the colonization rate is lower (3–6% and 2%, respectively), but still significant, with a CTX-M-type ESBL dominance [19,20]. The rate of colonization with carbapenemase-producing Enterobacterales is usually much lower (<10%). Unfortunately, it is also increasing worldwide, with some hotspots, for example, in China, with rates as high as 54% [20]. The import and spread of ESBL- or carbapenemase-producers has been recognized as a serious concern, and the travel to (sub)tropical regions (where colonization rates are known to be high) is a risk factor for acquiring colonization by the abovementioned resistant strains [19,21,22]. As there is a strong correlation between colonization with ESBL- and carbapenemase-producers and hospital-acquired infections (and the subsequent clinical consequences associated with these infections), attempts to control gastrointestinal colonization by MDR bacteria have received substantial attention in recent years [23]. These methods and techniques include heightened contact precautions with travelers returning from high-risk countries, selective decontamination, and administration of probiotics, phage preparations, or the use of fecal microbiota transplantation (FMT).

One of the main hindering factors associated with the attempts to eliminate gastrointestinal colonization is that the underlying mechanisms of developing intestinal carriage and clearance of MDR pathogens are not well understood. Apart from inter- and intra-individual differences in colonization risk (underlying conditions, immunosuppression, gut health), lifestyle factors, nutrition, and medicines are also known to influence microbiota composition [24,25,26,27]. Antibiotics (especially broad-spectrum agents) are known to alter gut microbiota composition and, in turn, create favorable conditions for the overgrowth of MDR or opportunistic bacteria, leading to infection [19,25,27,28]. In addition, exposure to antibiotics often leads to the upregulation and rapid spread of antibiotic resistance genes among the members of the gut microbiota via horizontal gene transfer (HGT), leading to the exacerbation of resistance rates [29]. While the administration of antibiotics is a known risk factor for colonization with an MDR strain, their underlying role in the colonization is still unclear. In our previous study, we identified that antibiotic treatment not only affected the degree of gut colonization with an ESBL- and OXA-162 carbapenemase-producing *K. pneumoniae* (ECKP) in mice but also the copy number of the resistance genes located on the conjugate plasmid [30].

As gut colonization with MDR strains is a growing clinical concern, mechanistic studies aiding the prospects of decolonization are a priority. The aim of our present study was to determine the underlying changes in the gut microbiota to shed more light on our previously published results highlighting that antibiotic treatments may have strong effects, negative or even positive, on the gastrointestinal colonization with ECKP on clone and resistance gene levels, and to ascertain how they relate to our observations. To this end, metataxonomic analyses of the gut microbiota dynamics were carried out during colonization with ECKP in mice. Furthermore, the effects of antibiotic administration (ampicillin, ceftazidime, and ciprofloxacin) on the establishment and elimination of ECKP intestinal colonization were also assessed.

## 2. Results

### 2.1. Fecal Microbiota Diversity during the Different Antibiotic Treatments after Colonization with ECKP

To assess colonization dynamics and the effects of antibiotic treatments, ampicillin pre-treated mice were administered the ECKP isolate *K. pneumoniae* KP5825 via oral gavage. Following colonization, ampicillin, ceftazidime, ciprofloxacin, or no antibiotics (to serve as a control group) were administered to the animals’ drinking water. With the aim of analyzing microbiota characteristics in the various treatment groups and the relationships between the KP5825 strain and the gut microbiota, stool samples were collected from each mouse in each group.

Microbiota composition was surveyed by sequencing the 16S rRNA gene from the fecal pellets. Miseq sequencing generated 218,894 reads per sample on average between 147,173 and 287,791 reads (standard deviation: 35,049). We assumed that the gut microbiota of the mice in different groups were similar before the antibiotic treatment. Comparative analyses further revealed distinct compositional differences of gut microbiota, depending upon the nature of various antibiotic treatment regimens.

**Figure 1:**
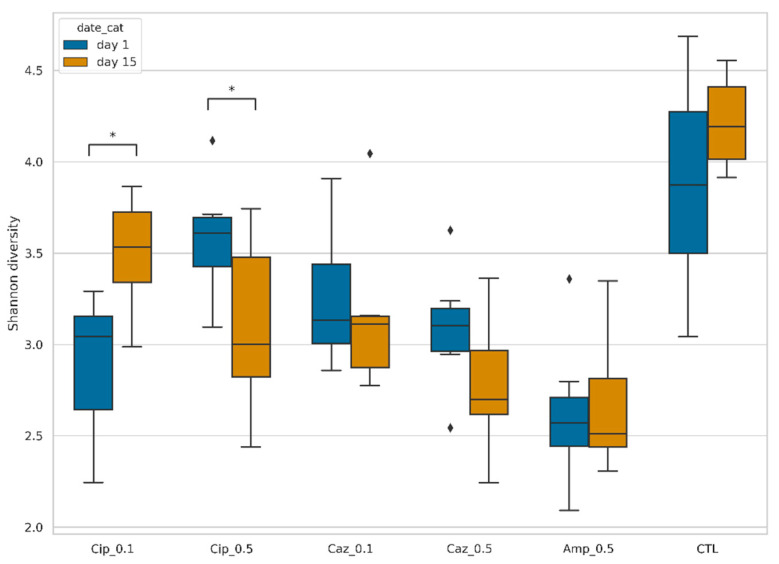
Shannon diversity of fecal samples, organized by sampling date and type of treatment. Box plots show the distribution of diversities in each group. Asterisks (*) show changes with *p* < 0.05 set as the limit for statistical significance. Diamonds (◊) shows the outlier values.

The bacterial diversity of each treatment group was calculated on the first and the fifteenth days and expressed using the Shannon index. The bacterial diversity was the highest in the control (CTL) group. During the observed period, bacterial diversity increased markedly in the Cip_0.1 group (*p* = 0.01729). The bacterial diversity was decreased for the fifteenth day in Caz_0.1 (*p* = 0.262618), Caz_0.5 (*p* = 0.123485), and Cip_0.5 (*p* = 0.04995) groups as well (Figure 1).

### 2.2. Alterations in Gut Microbiota Composition during the Different Antibiotic Treatments after Colonization with ECKP

**Figure 2:**
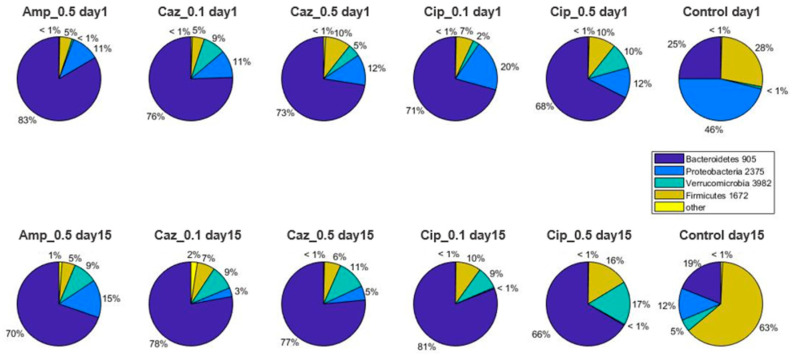
Phylum level abundances of bacteria. Average values of relative abundances at the phylum level were calculated for samples from the same treatment groups at both time points.

**Figure 3:**
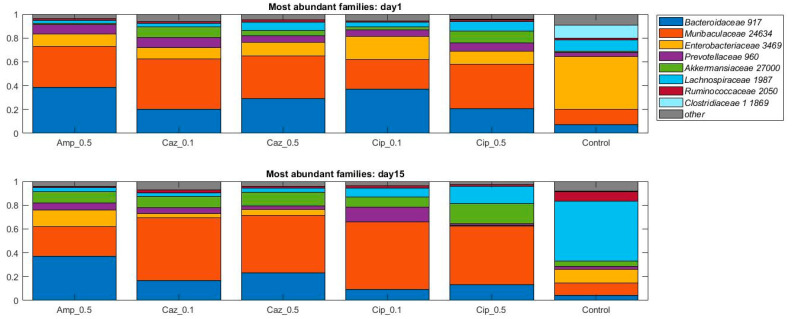
Relative abundances of the eight most abundant taxonomic families in the dataset over time. Average values of relative abundances at the family level were calculated for samples from the same treatment groups. Elements are shown if they have at least 5% relative abundance in at least one of the averaged samples.

**Figure 4:**
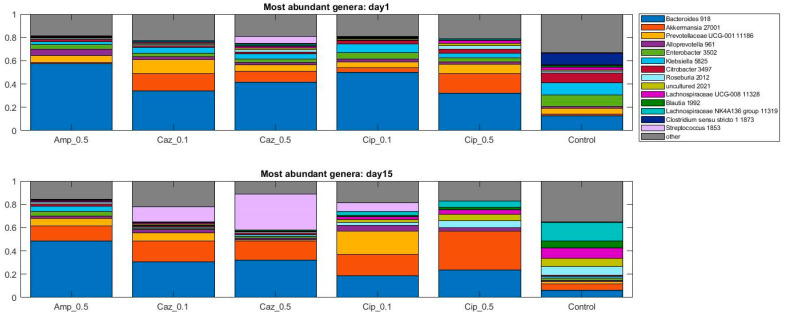
Relative abundances of the fourteen most abundant genera in the dataset over time. Average values of relative abundances at the genus level were calculated for samples from the same treatment groups. Elements are shown if they have at least 5% relative abundance in at least one of the averaged samples.

Comparative analyses further revealed distinct compositional differences in gut microbiota, depending upon the various antibiotic treatment regimens. Characteristic proxy descriptors for the effects of antibiotic treatment on the stool microbiota are shown in Figure 2, Figure 3 and Figure 4. At phylum level (Figure 2), Proteobacteria in the Cip_0.1 group decreased from 20% to ~0% (*p* = 0.0006); during high-dose ciprofloxacin treatment in the Cip_0.5 group, it decreased from 12% to ~0% (*p* = 0.0006), whereas in the CTL group, Proteobacteria also decreased from 46% to 12% (*p* = 0.007) after two weeks. In the Firmicutes phylum, an increase was detected in the Cip_0.5 group (10 vs. 16%, *p* = 0.053) and in the CTL group (28 vs. 63%, *p* = 0.053). Bacteroidetes decreased in the Amp_0.5 treatment group from 25% to 19%, on average (*p* = 0.0023). Verrucomicrobia increased in the Amp_0.5 (~0% vs. 9%, *p* = 0.004), Caz_0.5 (5% vs. 15%, *p* = 0.026), Cip_0.1 (2% vs. 9%, *p* = 0.007), and Cip_0.5 (10% vs. 17%, *p* = 0.011) groups, respectively.

When the analysis was performed at lower taxonomic ranks, more significant changes were identified in some other taxa. Effect of antibiotic treatment was observed on family level as well (Figure 3): an increase in bacterial abundance was identified in the *Lachnospiraceae* in the CTL group (10% vs. 50%, *p* = 0.0023) and in Cip_0.1 and Cip_0.5 groups (4% vs. 7%, *p* = 0.053, 8% vs. 14%, *p* = 0.053); however, a decrease was detected in the Caz_0.5 group (7% vs. 3%, *p* = 0.0262). Decreased abundance was recognized in Enterobacterales in the CTL group (44% vs. 11%, *p* = 0.007), Cip_0.1 and Cip_0.5 groups (19% vs. 0%, *p* = 0.0006, 11% vs. 0%, *p* = 0.0023). At the genus level, an average increase of bacterial abundance was identified in the *Streptococcus* genus in the ceftazidime treatment groups (Caz_0.1 and Caz_0.5) from the first to the fifteenth day. However, the greatest increase not only on the relative but also on the absolute level was observed in the Caz_0.5 group (6% vs. 30%, *p* = 0.1189) (Figure 4). The phylum, family, and genus level taxonomic composition of each sample may be found in Supplementary Figures S1–S3 in the Supplementary Material S1.

### 2.3. Correlation between Diversity and Taxonomy

**Figure 5:**
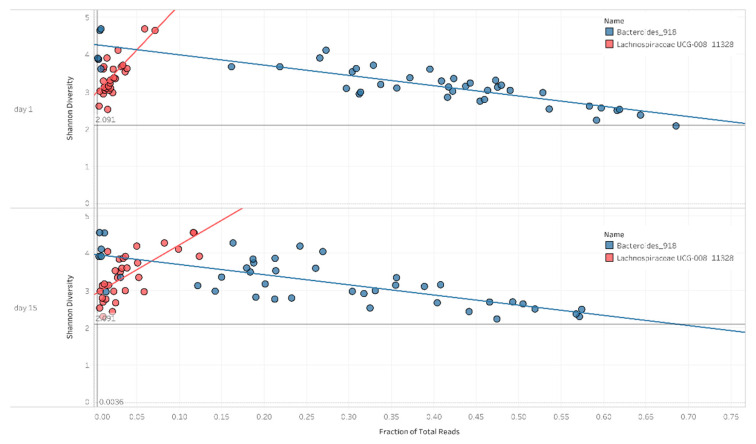
Scatter plot for Shannon diversity and bacterial abundance on day 1 and day 15. The figures present a positive and negative correlation between the Shannon diversity and the abundance of *Bacteroides* and *Lachnospiraceae* UCG-008, as well as the fitted trend lines.

During the analysis of the diversity and taxonomic composition of the samples, it was shown that the ratios of *Bacteroides* and *Lachnospiraceae* appear to play a major role in the decline in diversity. In the case of a high *Bacteroides* ratio, lower diversity values were observed, whereas in the case of a high *Lachnospiraceae* ratio, higher diversity values were observed. The high *Bacteroides* ratio had an adverse effect on diversification (*p* < 0.0001, r^2^ = 0.75). In contrast, *Lachnospiraceae* has had a beneficial effect on diversification (*p* < 0.0001, r^2^ = 0.5) (Figure 5).

### 2.4. Microbiota Changes Corresponding to Individual Treatment during Colonization

**Figure 6:**
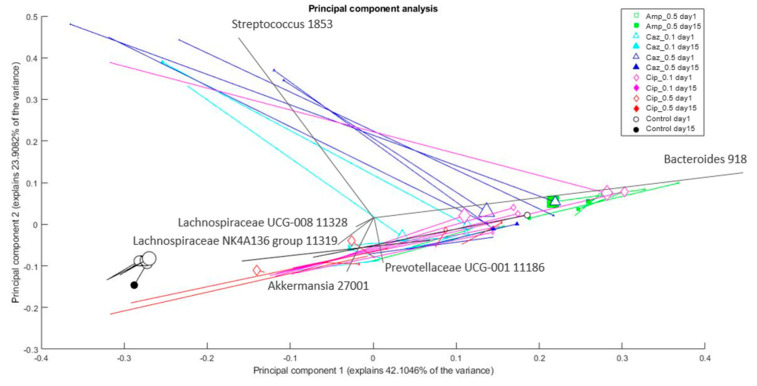
Changes in the composition of bacterial genera in the microbiota after different treatments. The % of variance explained by the component is shown on the axes; each marker represents one sample; colors correspond to the treatment (drug + dose) (Amp_0.5: green; Caz_0.1: cyan; Caz_0.5: blue; Cip_0.1: magenta; Cip_0.5: red; control: black); marker shapes correspond to the treatment (drug) administered. Legend: square: Amp; triangle: Caz; rhombus: Cip; circle: control; marker area refers to the sampling date (empty marker: day 1; full marker: day 15); lines connect the samples from the same source; therefore, changes in a sample (in the principal component analysis (PCA) space) can be seen from the empty marker to the full one following the line between them. Marker size correlates with *Klebsiella* content (relative abundance) of the sample. Arrows show the direction and importance of the original dimensions (the taxonomic categories) in the PCA space. PCA plot with uniform marker size (without *Klebsiella*) may be found in Supplementary Figure S4 in the Supplementary Material S1.

Changes in the composition of bacterial genera in the microbiota between the sampling points (corresponding to individual mice and different antibiotic treatment groups) were analyzed and visualized by principal component analysis (PCA). The treatment group of each individual mouse and the amount of *Klebsiella* is depicted in Figure 6. The CTL group is the most distinct. The Cip_0.1 group is the closest to the CTL group, whereas the Amp_0.5 group is the most dissimilar to the CTL group. The groups are separated mostly based on their *Bacteroides* content, which generally tends to drop by day 15. Among different treatment groups, the Amp_0.5 group members had the most, whereas CTL group members have had the least *Bacteroides* content. Most samples showed higher ratios for *Akkermansia* and *Lachnospiraceae* by day 15. Some samples (mainly from the Caz_0.5 group) have shown an intensive increase for *Streptococcus* on day 15 compared to day 1. The ratio of *Klebsiella* in most samples tends to decrease by day 15. The results of the PCA analyses on the family level are shown in Supplementary Figures S4–S6 in the Supplementary Material S1.

### 2.5. Interaction Analysis between the Colonizing ECKP and other Genera of the Microbiota

The principal question of our study was the role of the microbiota in the colonization with ECKP; therefore, the relationship between *Klebsiella* and other bacteria of the microbiota was studied. Additionally, the influence of bacterial genera independent from *Klebsiella* colonization was also investigated. The correlation between *Klebsiella* and all the other genera within all the treatment groups was accessed with scatter plots for the day-15 samples. Comparative analyses further revealed distinct compositional differences in the gut microbiota among the different antibiotic treatment groups.

In the present study, a correlation was considered strong if the Spearman’s correlation coefficient was over 0.6 or below −0.6. Within these limitations, strong positive correlation was observed among *Klebsiella* and *Citrobacter* 3497 (Spearman’s rho: 0.966501, *p*-value: 3.066630 × 10^−259^), *Enterobacter* 3502 (Spearman’s rho: 0.911455, *p*-value: 5.092763 × 10^−17^), *Raoultella* 3521 (Spearman’s rho: 0.882772, *p*-value: 1.061797 × 10^−14^), *Pantoea* 3513 (Spearman’s rho: 0.869518, *p*-value: 7.965868 × 10^−14^), *Escherichia-Shigella* 3504 (Spearman’s rho: 0.842450, *p*-value: 2.659530 × 10^−12^), and *Serratia* 3523 (Spearman’s rho: 0.776160, *p*-value: 1.549006 × 10^−09^). *Klebsiella* and other bacteria of the *Enterobacteriaceae* family, such as *Citrobacter*, *E. coli/Shigella,* all demonstrated a positive correlation, which indicates that if there was an increase in the amount of *Klebsiella* in the microbiota, the levels of these previously mentioned strains also increased.

**Figure 7:**
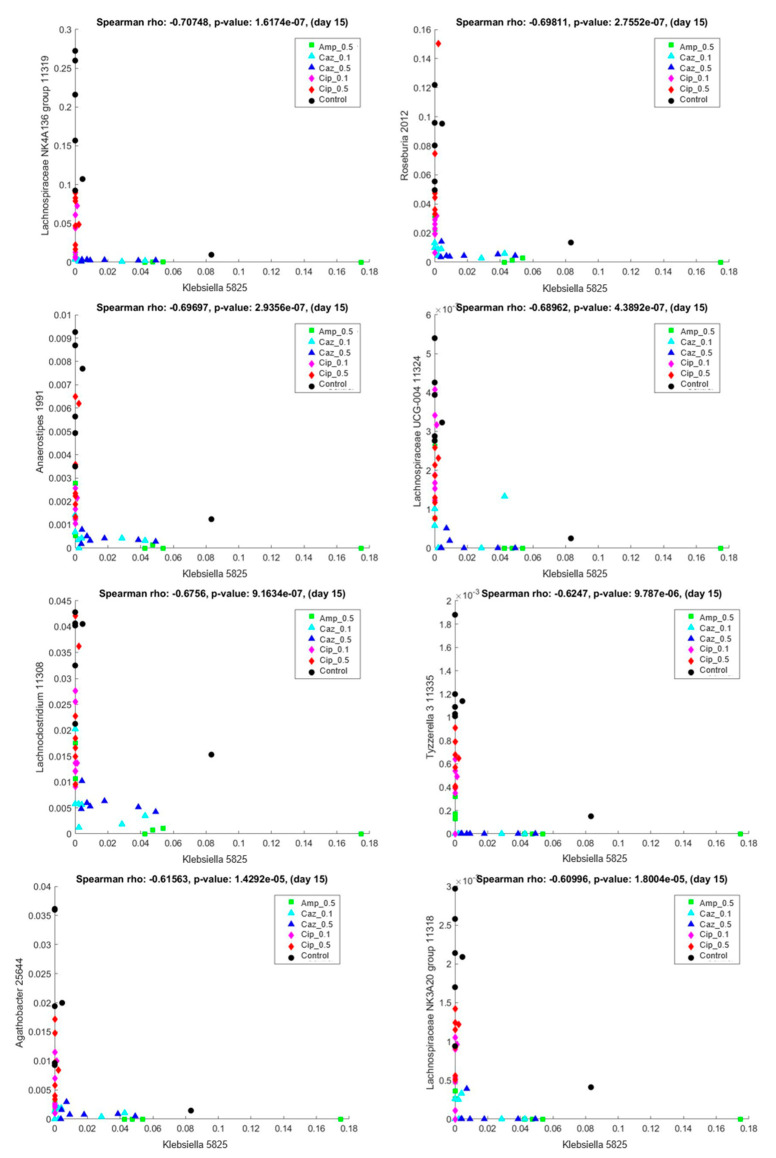
Correlation between *Klebsiella* KP5825 (x-axis) and other bacterial genera in samples of day 15. *Lachnospiraceae* NK4A136 group 11319, *Roseburia* 2012, *Anaerostipes* 1991, *Lachnospiraceae* UCG-004 11324, *Lachnoclostridium* 11308, *Tyzzerella* 11335, *Agathobacter* 25664, and *Lachnospiraceae* NK3A20 group 11318 (y-axis).

In contrast, a strong negative Spearman’s correlation of KP5825 was found with *Lachnospiraceae* NK4A136 group 11319 (Spearman’s rho: −0.707483, *p*-value: 1.617432 × 10^−7^), *Roseburia* 2012 (Spearman’s rho: −0.698115, *p*-value: 2.755227 × 10^−7^), *Anaerostipes* 1991 (Spearman’s rho: −0.696975, *p*-value: 2.935625 × 10^−7^), *Lachnospiraceae* UCG-004 11324 (Spearman’s rho: −0.689624, *p*-value: 4.389162 × 10^−7^), *Lachnoclostridium* 11308 (Spearman’s rho: −0.675595, *p*-value: 9.163409 × 10^−7^), *Tyzzerella* 3 11335 (Spearman’s rho: −0.624699, *p*-value: 9.787015 × 10^−6^), *Agathobacter* 25644 (Spearman’s rho: −0.615631, *p*-value: 1.429190 × 10^−5^), and *Lachnospiraceae* NK3A20 group 11318 (Spearman’s rho: −0.609958, *p*-value: 1.800413 × 10^−5^). Representative scatter plots are shown in Figure 7. These bacterial genera were nearly exclusively present with low levels of KP5825 and were present in the CTL and ciprofloxacin-treated samples, whereas KP5825 was more frequent in the ampicillin and ceftazidime treatment groups. Supplementary Table S1 in Supplementary Material S1 contains the complete list of pairwise correlations among KP5825 with Spearman’s rho and *p*-values.

### 2.6. Co-Occurring Clusters of Bacteria during Dynamic Changes in the Microbiota Associated with ECKP Colonization

**Figure 8:**
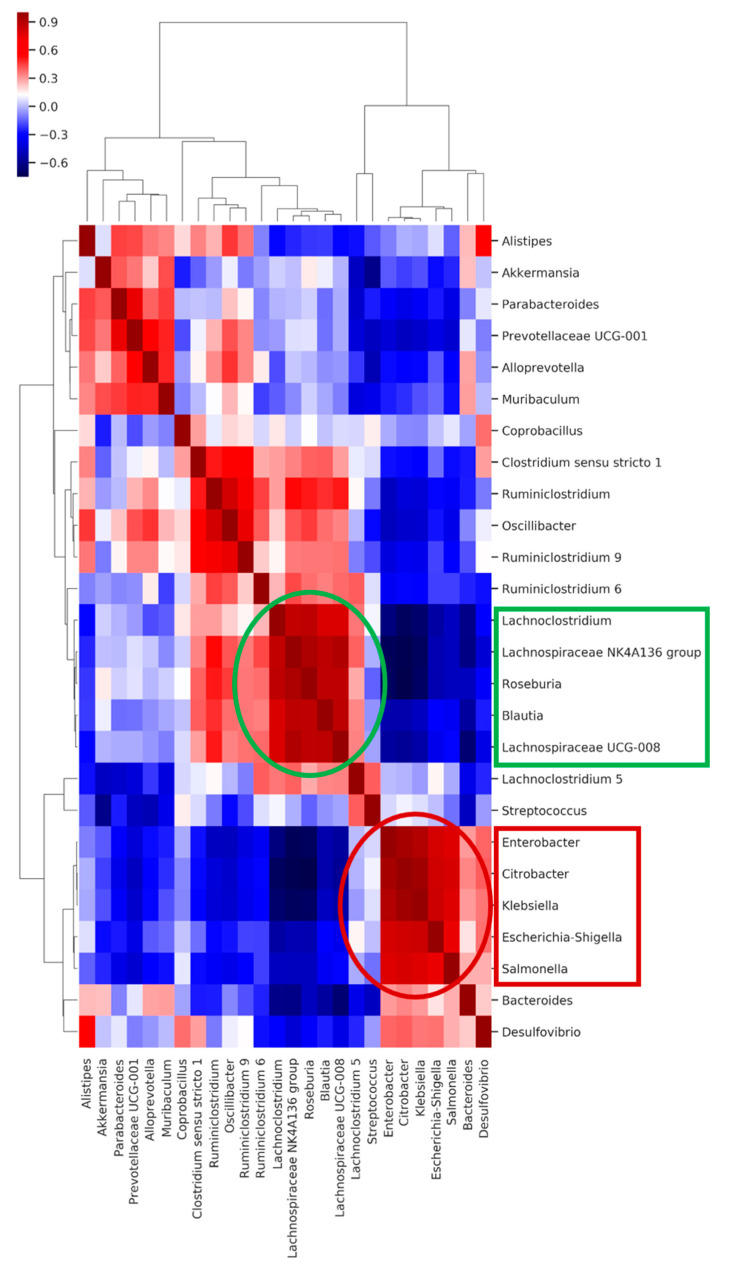
The heatmap shows the pairwise correlation (Spearman’s rho) between selected bacterial abundances in day-15 samples. The bacterial groups at the x- and y-axes were clustered based on Bray–Curtis distances. Colors are corresponding to the rho values: deeper blue colors represent stronger negative, deeper red colors stronger positive correlations.

The co-occurrence analysis between the colonizing *K. pneumoniae* strain and the different genera in the microbiota composition showed that the abovementioned genera have a strong positive or negative correlation with KP5825, indicating some direct or indirect negative exclusionary or symbiotic relationship between them. To further analyze the direct or indirect negative or positive interactions, a clustered heatmap was constructed from the samples and the bacteria that were represented in the feces in at least 1% (Supplementary Figure S7 in the Supplementary Material). The heatmap shows co-occurring clusters of frequent bacteria. The colonization rate of KP5825 showed an increase together with some genera, whereas it presented an inverse relationship with others. In order to identify the structure of these relationships in the studied microbiotas, correlations between the bacterial genera were also studied. Figure 8 shows the pairwise correlations (Spearman’s rho) between high bacterial abundances in day-15 samples. Two distinct bacterial groups could be observed: one group containing bacteria that changed their proliferation level at the same rate as the *Klebsiella* (consisting of *Enterobacter*, *Citrobacter*, *Salmonella,* and *Escherichia-Shigella* genera) and another bacterial group in which the trend of proliferation rate moved in the opposite direction (with *Lachnoclostridium*, *Roseburia*, *Blautia,* and *Lachnospiraceae* genera). The members of each cluster are strongly correlated, and they populate the same samples. However, there is a strong negative correlation between the two clusters, indicating that they have an exclusionary relationship with each other by some direct or indirect effect. Based on the cluster analysis, it seems that this cluster effect is stronger and more coherent than the individual bacterial abundances. The co-occurring cluster members can have a greater effect on the entire microbial community and also on the host than individual species.

## 3. Discussion

In our present study, the gut microbiota dynamics in mice were monitored during colonization with an ESBL- and OXA-162 carbapenemase-producing *K. pneumonaie* strain during various antibiotic treatments in order to ascertain critical factors in the microbiota dynamics that may aid the clearance of this MDR strain or lead to higher levels of colonization. Gut bacteria resistant to third-generation cephalosporins and carbapenems (most frequently *Klebsiella* spp. and *E. coli*) significantly contribute to the attributable deaths, sequelae, and disability-adjusted life-years in the burden of MDR bacteria [31]. These resistant isolates were previously prevalent in hospital-associated infections; however, their description in community-acquired infections is becoming more frequent [24,25,32]. In addition, in our globalized world, there is a substantial risk for the acquisition of ESBL-producing and/or CRE strains during travel to endemic areas, especially if one comes into contact with a hospital environment in these countries [21,26,27]. Clinical infections are often preceded by gut colonization by these MDR pathogens: the risk factors associated with the long-term fecal carriage of ESBL- and carbapenemase-producing *Klebsiella* and *E. coli* have been investigated previously in many clinical trials (associated with, e.g., travel history, proton pump inhibitor use) [21,24,33]. However, only very few studies have analyzed the relationship between the intestinal microbiota composition and carrier status [26,34]. Therefore, our study is of substantial importance in providing data on the dynamics and process of the colonization and the possible modifying factors, such as antibiotic exposure.

Natural or spontaneous clearance (also called spontaneous decontamination) of MDR bacteria from the gut microbiota is a well-known phenomenon; however, there is a substantial lack of knowledge regarding the underlying mechanisms hindering or facilitating this process [35]. In addition, there are large inter-individual differences observed in the time period required and the efficacy of decolonization after the exposure. However, it has been demonstrated that natural clearance occurs more rapidly in healthy individuals compared to hospitalized or immunosuppressed patients [36].

There is growing interest in developing methods for the decolonization of MDR bacteria from patients, both from clinical and infection control perspectives. Currently, available methods suggested for the clearance of MDR bacteria will be briefly discussed. The administration of probiotics was the first proposed method for the prevention and decolonization of MDR pathogens with controversial results [37]. Fecal microbiota transplantation (FMT; first used in the treatment of refractory *Clostridioides difficile* infections) was described as a potential strategy for decolonization. In the case of FMT, a complex microbial community is being transferred from one individual to another (cf. probiotics) in a controlled manner. Additionally, it has been postulated that FMT-administration has distinct effects on drug-resistant bacteria [38,39]. It may be presumed that various MDRs differ in their response to FMT, and the mechanism of resistance plays an important role in the response [40]. FMT presented with higher efficacy against MDR *Pseudomonas aeruginosa*, whereas lower success rates were observed in the case of ESBL-producing or New Delhi metallo-beta-lactamase 1-producing (NDM-1) *K. pneumoniae* [39,41,42]. In addition, during the FMT process, the donors may themselves carry drug-resistant bacteria, which may be transferred to the recipient, subsequently leading from the asymptomatic colonization to the development of severe, untreatable infections [43,44,45]. This issue has been recently highlighted due to the death of a patient, whose death was attributed to an infection caused by an ESBL-producing *E. coli* strain bacteremia, which was acquired by the patient during FMT [45]. Recently, with the emergence of the SARS-CoV-2 virus and the succeeding pandemic, the situation has become even more complicated, as the virus (not only viral RNA) is shed in the feces, and the uptake of the virus by enterocytes has been described; therefore, FMT also carries the risk of transferring the novel coronavirus to the recipient and the development of a subsequent nosocomial infection [46,47]. In addition, based on the report of Zao et al., distinct alterations in the gut microbiota occur in patients hospitalized due to COVID-19, irrespective of receiving antibiotic therapy or not, Whereas in patients receiving antibiotics, the number of symbiotic bacteria in the gut (e.g., *Faecalibacterium prausnitzii, Lachnospiraceae* spp., *Eubacterium rectale, Ruminococcus obeum,* and *Dorea formicigenerans*) decreased, the number of opportunistic pathogens (*Actinomyces viscosus, B. nordii, C. hathewayi*) increased in patients not receiving antibiotics. Zao et al. has shown that gut dysbiosis, a consequence of COVID-19 disease, persists after the microbiological and clinical cure has been reached. Consequently, a SARS-CoV-2 infection may increase the susceptibility of patients to become colonized by MDR bacteria (especially in a hospital environment) through dysregulation of the microbial flora of the gut [48].

The use of phages has also been suggested as a viable decolonization strategy, although, currently, there is only one report available detailing a case of successful treatment by a custom-made, lytic bacteriophage preparation, eliminating colonization with an MDR, KPC-3 carbapenemase-producing *K. pneumoniae* isolate [49]. Given that there currently is no adequate way to treat colonization with MDR bacteria, it becomes particularly important to understand the dynamic process of colonization in order to use the knowledge obtained in developing a decolonization strategy. Thus, our findings in this study may provide insight into designing complex and efficient decolonization strategies in the future. The results of our study support the concept that the intestinal microbiota affects the clearance of colonizing MDR bacteria, modeled by the ECKP strain in our mice experiments.

These results are in line with the observations performed in mice in which specific operational taxonomic units (OTUs) were shown to be associated with clearance of the MDR strains [50,51]. We have found that the phyla Bacteroidetes and Firmicutes were generally most dominant in all of the treatment groups; however, differences in their ratios were observed after the subsequent antibiotic treatments; Bacteroidetes was more common in the groups treated with antibiotics, compared to the control group. Notably, we observed a significant difference between the different antibiotic-treated groups in beta but not alpha diversity, implying that the difference is the relative abundance of some bacterial community members. In contrast, in a study by Piegwam et al. assessing the microbiota of carrier and non-carrier patients of ESBL-producing Enterobacteriaceae, conflicting results were found: the phylum Bacteroidetes (in particular, *B. uniformis*) was significantly more abundant in non-carriers [52].

The detrimental effects of antibiotic therapy on the intestinal microbiota have been well documented. Administration of these drugs leads to the decrease in the relative abundance of microbes; in addition, they may have detrimental effects on the transitory resistome of the gut, increasing the copy number of resistance genes in MDR isolates and facilitating HGT [16,29,53,54]. Based on our previous findings, ampicillin and ceftazidime treatment increased the colonization of the ECKP strain and the copy number of the CTX-M-15 resistance gene that was resident in the plasmid of KP5285. In contrast, ciprofloxacin did not increase the colonization rate or the expression level of the examined resistance genes [30]. The role in the increasing frequency of gastrointestinal colonization by ESBL and carbapenemase-producers of the worldwide reduction of fluoroquinolone use is debatable, whereas the use of β-lactam antibiotics has increased globally [4]. As we have seen from our previous results, the use of these drugs may play a role not only in maintaining colonization but also in increasing the copy number of resistance genes. However, the effects of antibiotic treatment in the context of affecting colonization have not been extensively studied. An important finding of our study is that the composition of the microbial community plays a primary role in the degree of colonization with MDR bacteria and the antibiotic susceptibility of the individual MDR strain affects this process to a lesser extent.

This is supported by the fact that ciprofloxacin treatment dose-dependently reduced colonization of the ECKP strain to a much greater extent than in the antibiotic-free group, despite the fact that the KP5825 strain showed a high level of resistance to ciprofloxacin [30]. Based on our findings, the treatment with antibiotics has led to complex changes in microbiota composition, not only as a direct function of antibiotic treatment, but as the association of distinct bacterial communities. Another significant result of this study is the detection of these well-defined microbial clusters within the intestinal microbiota of mice and their association with the clearance of the carbapenemase-producing strain. Indeed, it was observed that a set of bacteria from the *Lachnospiraceae* family (including *Agathobacter*, *Anaerostipes*, *Lachnoclostridium* 11308, *Lachnospiraceae* UCG-004, *Lachnospiraceae* NK3A20 group 11318, *Lachnospiraceae* NK4A136 group 11319, *Roseburia,* and *Tyzzerella*) showed an inverse relationship with the carriage rate of the ECKP strain; these species were more abundant in mice who showed significantly decreased carriage rates of the carbapenemase-producer. Species from the *Ruminococcaceae*, *Lachnospiraceae*, and *Veillonellaceae* families are associated with the normal gut of humans and other mammals, but they are also involved in the production of short-chain fatty acids (SCFAs); these SCFAs are known to inhibit the growth of several species belonging to the Enterobacteriaceae family [55,56,57]. Based on a recent study by Sorbara et al., *Lachnospiraceae* isolates are likely to impact colonization resistance through the acidification of the intestinal lumen and the expression of lantibiotics (the latter being important in inhibiting the growth of competing bacteria) [56,58]. They were also shown to influence the host mucosal immune cells and enterocytes via the production of butyrate, and they may contribute to the synergistic mechanism of cognate microbial families within their cluster by heterogeneous polysaccharide metabolism. The underlying suspected mechanism is that at low pH in the gut, butyrate will penetrate through the bacterial membrane and has a direct killing effect on the members of the *Enterobactericeae* family [56,59]. However, the administration of antibiotics in the gut leads to a decrease in pH levels; therefore, the produced butyrate will lose its bactericidal effects [56].

On the other hand, species of the Enterobacteriaceae family showed a strong positive correlation with the amount of KP5825 in the Spearman’s-correlation matrix. Their changes occurred in parallel; thus, the increase or decrease of the MDR-*Klebsiella* strain was also associated with the subsequent increase or decrease in the amount of other Enterobacteriaceae, such as *Citrobacter*, *Enterobacter,* or *Escherichia-Shigella*. Members of the Enterobacteriaceae family possess distinct mechanisms to promote each other’s growth through the generation of inflammatory processes and dysbiosis. If the amount of butyrate-producing bacteria decreases, it may lead to an increased expression of inducible nitrogen-oxide synthase (iNOS) in epithelial cells. Byproducts of iNOS can be used only by the *Enterobacteriaceae* during nitrogen fermentation; thus, instead of a competitive relationship between taxonomically-related species, this results in additional proliferation of the cluster including Enterobacteriaceae members, leading to a vicious circle, a positive feedback loop [60]. Another possibility to consider is co-colonization with multiple MDR bacteria, which is presumably also influenced by shifts in microbiota dynamics. In our studies, mice colonized in a greater extent with MDR-*Klebsiella* also had a much higher proportion of *Streptococcus*, especially in samples in the high-dose ceftazidime treatment group, as we observed earlier [30]. This phenomenon was observed in both individual and pooled group evaluations. Similar observations have been described by Collingwood et al. during the co-colonization of intensive care unit (ICU) patients with carbapenem-resistant *Klebsiella* and vancomycin-resistant enterococci (VRE) [28]. Their results also suggested that VRE and *K. pneumoniae* interact in the fecal microbiota. Importantly, the differences in the microbiota between the groups seem to be driven by *Klebsiella* and *Enterococcus* themselves, with a higher abundance of a *Klebsiella* OTU in *Klebsiella* and co-colonized groups and a higher abundance of an *Enterococcus* OTU in VRE-colonized groups compared with the non-colonized group [28].

**Figure 9:**
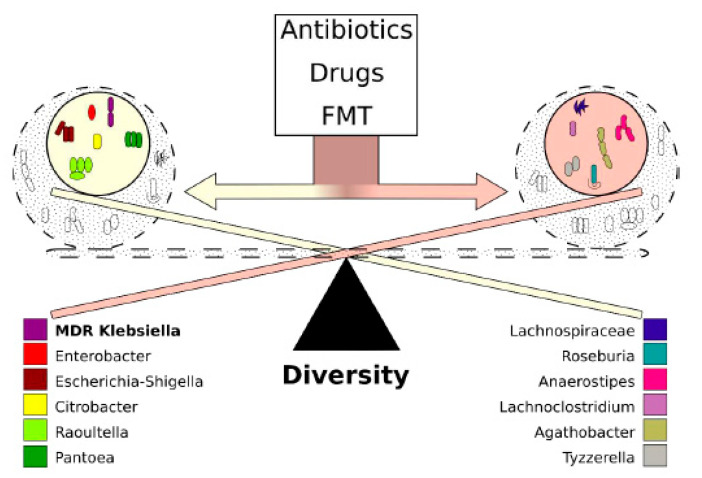
The dynamics of colonization with MDR *K. pneumoniae.* Colonization dynamics with multi-drug-resistant *K. pneumoniae* are determined by the behavior of microbiota-cluster groups rather than the individual vulnerability of the bacteria. One of the dominant clusters contains bacteria belonging to the *Enterobacteriaceae* family, whereas the other dominant cluster contains *Lachnospiraceae* and other short-chain fatty acid-producing bacteria. Bacteria belonging to the same cluster reinforce each other, whereas the contrasting clusters act against each other. The effect of clusters overrides the effect of individual bacteria. The balance between clusters is mostly affected by the use of antibiotics, other drugs, and fecal microbiota transplantation (FMT).

Our results point out that a change in the perspectives regarding future decolonization strategies is needed, one that considers the systems disorder occurring in gut microbiota dynamics after colonization with an MDR species. In addition, the effects of antibiotics on the microbiota composition and the corresponding beneficial or detrimental changes in gut bacteria affecting the capacity of the host to eliminate colonizers must be taken into consideration. We have shown that distinct bacterial families have associated into microbial clusters (most notably, members of *Lachnospiraceae* and *Enterobacteriaceae*), collecting taxonomically close species around themselves to produce survival benefits in the gut microbiota. These associations between different species do not develop at random. These may be attributed to the presence of specific metabolomic networks. Interestingly, members of *Enterobacterieaceae* and the colonizing KP5825 strain have shown a correlational relationship, whereas *Lachnospiraceae* and related organisms were more abundant in mice, where the rate of the ECKP was lower. As demonstrated by our results, the two microbial clusters have contrasting effects on the colonization rate of the carbapenemase-producer, and the effect of administered antibiotics on the ECKP colonization may also be attributed to the effects on these clusters. Thus, new modifiers of microbiota composition (which may already be present in smaller quantities in the host) may hold the promise of effective decolonization treatments. In the future, tailor-made metabolomic cocktails (containing substrates and end-products deliberately chosen) may hold the key to propagating advantageous or suppressing disadvantageous clusters, facilitating natural clearance of MDR pathogens from the gut. In a study by Leo et al., the proportion of *Enterobacteriaceae* in the microbiota of FMT-treated MDR carriers was significantly lower than the baseline, whereas the abundance of *Bifidobacterium* species, together with butyrate- and propionate-producing species from *Lachnospiraceae* and *Ruminococcaceae* families, were significantly enriched after treatment [61]. Thus, it may be concluded that FMT, even with the recently described safety concerns, is the closest thing we currently have that considers all the abovementioned points: during fecal transplantation (in contrast to the administration of probiotics, where only one or few species are introduced) a complex community or cluster or bacteria and the corresponding metabolomic network is being transferred into the host, which cannot be produced yet by artificial means. The presence of these networks in the FMT may be crucial in shifting the ecological conditions in the gut microbiota to restore gut health and favor the elimination of MDR-colonizing species (Figure 9).

## 4. Materials and Methods

### 4.1. Study Design, Bacterial Strains

**Figure 10:**
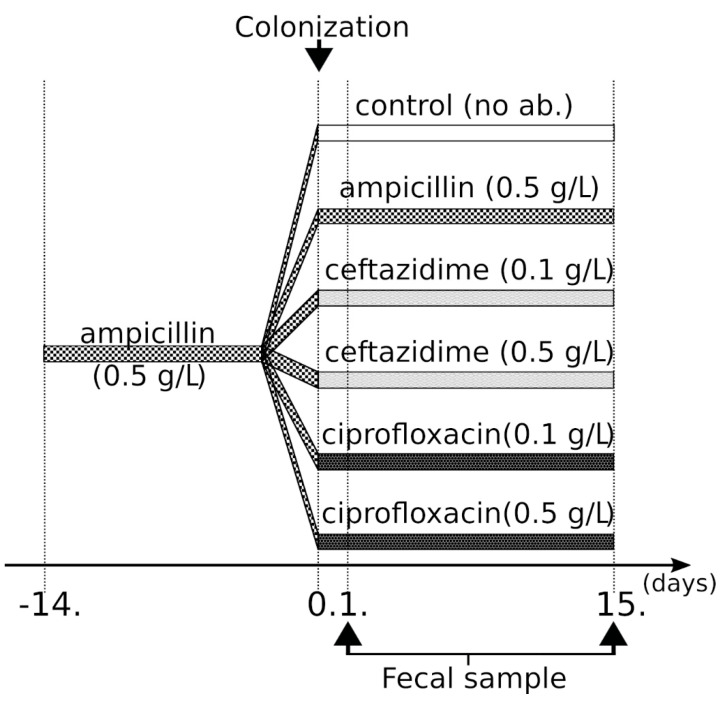
Colonization protocol used in our experiments.

The *K. pneumoniae* KP5825 isolate used in our study produced the CTX-M-15 ESBL and OXA-162 carbapenemase enzymes (ECKP), in addition to containing numerous antibiotic resistance genes: for β-lactam resistance: *bla*_SHV-28_, *bla*_OXA-1_, *bla*_OXA-162_, and *bla*_CTX-M-15_; for aminoglycoside resistance: *aac(3)-IIa, aph(3’)-Ia,* and *aac(5’)Ib-cr*; for fluoroquinolone resistance: *aac(6’)-Ib-cr, oqxA,* and *oqxB*. During colonization experiments, 5 × 10^6^ CFU of *K. pneumoniae* KP5825 was administered by oral gavage in a volume of 200 µL on the fourteenth and fifteenth days of ampicillin pretreatment. After the colonization, 0.5 g/L ampicillin (Amp_0.5), 0.1 g/L ceftazidime (Caz_0.1), 0.5 g/L ceftazidime (Caz_0.5), 0.1 g/L ciprofloxacin (Cip_0.1), 0.5 g/L ciprofloxacin (Cip_0.5), or no antibiotic (control group; CTL) was further administered to the animals (seven mice in each group) in the drinking water for two weeks and changed every day (Figure 10). Mice were single-housed at the time of the colonization experiments. Animals were maintained in a specific pathogen-free facility at the Institute of Medical Microbiology, Semmelweis University. All mouse handling, cage changes, and fecal pellet collection were performed in a biosafety level 2 (BSL-2) facility wearing sterile gowns, masks, and gloves. Animals were maintained and handled in accordance with the recommendations of the Guidelines for the Care and Use of Laboratory Animals.

### 4.2. Microbiota Analysis

To investigate the gut microbial characteristics in the different antibiotic treatment groups, as well as the relationships between the gut microbiota and colonization rate with KP5825, stool samples were collected from each mouse in each group; altogether, *n* = 84 fecal samples were collected. Microbiota analysis was completed for seven mice per group at each time point (on the first and fifteenth day) by deep-sequencing bacterial 16S rRNA gene amplicons. Briefly, DNA was extracted from ~80 mg of feces using the QIAamp PowerFecal DNA kit (Qiagen; Frederick, MD, USA) according to the manufacturer’s instructions with lysis conditions optimized to increase the ratio of nonhuman to human DNA. The V3–V4 region of the bacterial 16S rRNA gene, considered as the most promising for bacterial deep-sequencing-based diversity. Thus, a primer pair was designed for this region and was PCR amplified. Single amplicons of ~460 bp were cleaned up using AMPure XP beads (Agencourt AMPure XP) and visualized by DNA 1000 kit with Agilent 2100 Bioanalyzer. Dual indices (barcodes) and Illumina sequencing adapters were added to the amplicons using the Nextera XT Index kit (Illumina, Inc., San Diego, CA, USA), followed by DNA purification (Agencourt AMPure XP). Individual barcoded DNA samples were then quantified (Qubit dsDNA HS Assay kit with Qubit 2.0; Thermo Fisher Scientific), qualified (DNA 1000 kit with Agilent 2100 Bioanalyzer), normalized, and pooled. Multiplexed libraries were diluted to 13 pM and denatured with NaOH prior to sequencing on the MiSeq system (Illumina) using the MiSeq reagent kit v3 600 cycle (2 × 300 bp; Illumina).

### 4.3. Metataxonomic Analysis

The demultiplexed raw sequencing data were retrieved from the Illumina BaseSpace. The quality control (QC) of the raw reads was carried out by FastQC and MultiQC [62]. After the QC, low-quality reads were trimmed by Trimmomatic [63]. The first 12 base pairs were removed, as well as the consecutive low base calls (Phred score ≤ 20), and only reads with a minimal length of 36 were kept. In our analysis, the small subunit (SSU) Ref NR 99 database was used, downloaded on 22/03/2019 (release 132) of SILVA [64]. This contains non-redundant sequences (identity threshold is 99%). The database FASTA file was preprocessed, and the RNA sequences were converted by replacing the uracils (U) with thymines (T). The preprocessed database was indexed by the Kraken2 tool (kraken2-build) with k-mer = 31. Kraken2 aligned and classified the short-reads, and the final estimation of the taxon abundances in various taxonomic levels was conducted with Bracken (with threshold = 20 for the minimal number of reads required for classifying categories) [65].

### 4.4. Statistical Analysis

In order to quantify the change between the microbiota compositions for each mouse in the downstream analysis, the taxa with very low abundance (hit count < 50) were discarded, and also those that are not present in any samples at least with the proportion of 1%.

The Shannon index was used as the alpha diversity measure for the treatment groups. The statistical significances between the studied groups were estimated with the Wilcoxon rank-sum test. The relative abundance plots show only those taxonomic categories that were present with at least 5% relative abundance in at least one of the sample groups averaged by treatments. Statistical significance values for the changes between time points were calculated again by the Wilcoxon rank-sum test. Spearman’s correlation was calculated between the read ratios of selected frequent bacterial genera and the Shannon diversity index of the corresponding samples. The Shannon index and the read ratios were visualized in scatter plots, and linear lines were fitted to show the trends of correlations. Principal component analysis (PCA) was performed in multiple taxonomic levels, based on the relative abundance values, and the samples were plotted in the new space defined by the two strongest principal components (explaining the most from the inter-sample variance). The orientations of the original taxonomic categories contributing the most to those components were also visualized in this space.

Spearman’s correlation coefficients were calculated between *Klebsiella* and other bacterial genera in each of the samples on day 15. Pairs of the strongest positive and negative correlations (|rho| ≥ 0.4 and *p*-value < 0.05) were listed, and a selection of them (rho < −0.6) were visualized in scatter plots. A heatmap was generated from the read ratios of genera that were represented in at least 1% in the samples in order to identify samples with similar compositions. A selection of frequent genera were chosen from this list, and between these genera, pairwise Spearman’s correlations were calculated. The rho values of these correlations were clustered again in another heatmap to identify strongly correlated co-appearing groups of genera with special attention to *Klebsiella*. The heatmaps were clustered based on the Bray–Curtis distance measure. The calculations of the Shannon indices, the heatmaps, and the correlation analysis were performed with the scikit-bio package [http://scikit-bio.org/docs/0.5.5/index.html] (version 0.5.5) of Python. The relative abundance plots, scatter plots, and the PCA analysis was carried out in MATLAB (version R2017b).

## 5. Conclusions

The carriage of drug-resistant bacteria in the gut microbiota may lead to serious infections in vulnerable patient populations; therefore, studies on innovative and comprehensive decontamination strategies should be highlighted. Our experimental results suggest that a new concept, a new way of thought should be introduced in designing future endeavors for MDR decolonization. In conventional microbiological studies on colonization, the individual features and antibacterial susceptibility of bacteria are studied; however, the composition of them and the effects of the microbiotic environment surrounding MDR bacteria have not been extensively studied. In the future, colonization studies may need to be supplemented by the study of the host community composition and identifying bacterial clusters that may be capable of suppressing or enhancing the invader species. From the currently known decolonization strategies, fecal transplantation is the most successful treatment option so far, which was further underlined by our experiments, providing mechanistic insight into a possible mechanism of action, even if the clearance rate is not too high. The fact that FMT is the most successful treatment available confirms our perspective that we should think in bacterial groups (as complex networks of taxonomical and metabolomic interactions) rather than individual bacteria.

## Data Availability

The datasets supporting the conclusions of this article are included within the article and its additional files. The sequencing data generated during the current study were submitted to NCBI’s Sequence Read Archive (SRA) and are accessible with SRA accession number PRJNA595721.

## Supplementary Materials

The following are available online at https://www.mdpi.com/2079-6382/10/3/268/s1. **Figure S1.** Relative abundances at phylum level for each mouse feces sample on day 1 and day 15 that have at least 5% relative abundance in at least one of the samples. **Figure S2.** Relative abundances at family level for each mouse feces sample on day 1 and day 15 that have at least 5% relative abundance in at least one of the samples. **Figure S3.** Relative abundances at genus level for each mice feces sample on day 1 and day 15 that have at least 5% relative abundance in at least one of the samples. **Figure S4.** Principal component analysis of the different treatments and the changes in the composition of bacterial genera in the microbiome of each mouse in time. **Figure S5.** Principal component analysis of the different treatments and the changes in the composition of bacterial families in the microbiome of each mouse in time. **Figure S6.** Principal component analysis of the different treatments and the changes in the composition of bacterial families in the microbiome of each mouse in time. **Figure S7.** Correlation between *Klebsiella* and other genera. Proliferation rates of bacteria from the stool samples of each mouse addressed to different treatments at different time points. **Table S1.** Pairwise Spearman’s correlations between *Klebsiella* and other bacterial genera with |rho| ≥ 0.4 and *p*-value < 0.05.

## Abbreviations

**Amp**	ampicillin
**Amp_0.5**	0.5 g/L ampicillin
**BSL**	biosafety level
**Caz**	ceftazidime
**Caz_0.1**	0.1 g/L ceftazidime
**Caz_0.5**	0.5 g/L ceftazidime
**CFU**	colony-forming unit
**Cip**	ciprofloxacin
**Cip_0.1**	0.1 g/L ciprofloxacin
**Cip_0.5**	0.5 g/L ciprofloxacin
**CRE**	carbapenem-resistant Enterobacterales
**CTL**	control group
**DALY**	disability-adjusted life years
**ECKP**	ESBL- and carbapenemase-producing *Klebsiella pneumoniae*
**EBSL**	extended-spectrum β-lactamase
**FMT**	fecal microbiota transplantation
**HGT**	horizontal gene transfer
**ICU**	intensive care unit
**iNOS**	Nitric oxide synthase
**KPC**	Klebsiella pneumoniae carbapenemase
**MDR**	multidrug-resistant
**NDM**	New Delhi metallo-beta-lactamase
**OUT**	operational taxonomic unit
**PCA**	principal component analysis
**PCR**	polymerase chain reaction
**QC**	quality control
**SARS-CoV-2**	Severe acute respiratory syndrome coronavirus 2
**SCFA**	short-chain fatty acid
**SSU**	small subunit
**T**	thymine
**U**	uracil
**WRE**	vancomycin-resistant enterococci
**WHO**	World Health Organization
