# Supplementary material for: Colonization Dynamics of Multidrug-Resistant Klebsiella pneumoniae Are Dictated by Microbiota-Cluster Group Behavior over Individual Antibiotic Susceptibility: A Metataxonomic Analysis
Source: Antibiotics (Basel). 2021 Mar 7;10(3):268. doi: 10.3390/antibiotics10030268 (PMC8001907; doi:10.3390/antibiotics10030268)
Supplement: Supplementary file 1 [file antibiotics-10-00268-s001.pdf]

Supplementary Material S1

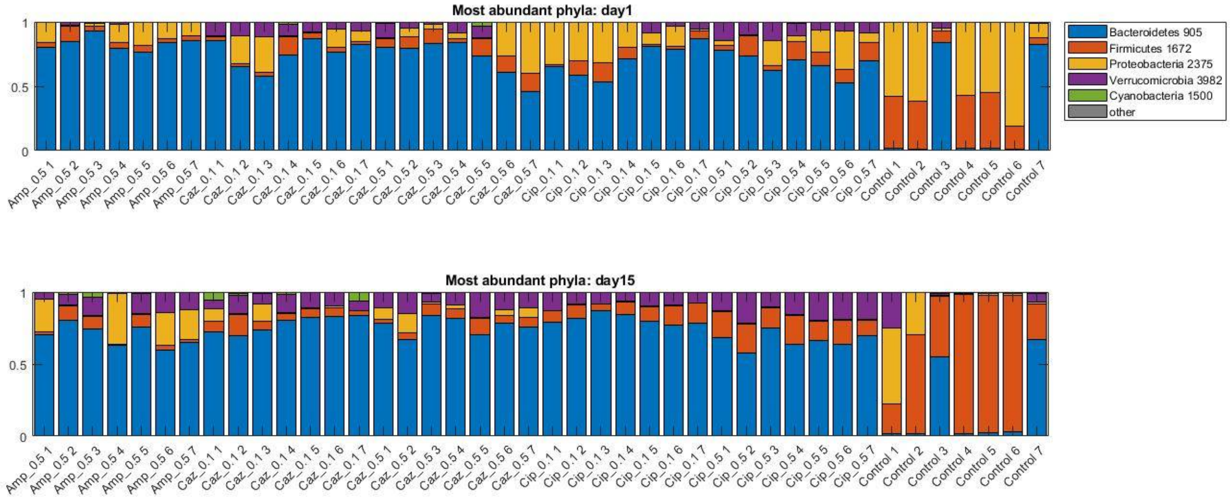

Figure S1. Relative abundancies at phylum level for each mice feaces sample on day 1st and day 15th that have at least 5% relative abundancy in at least one of the samples.

Supplementary Material S1

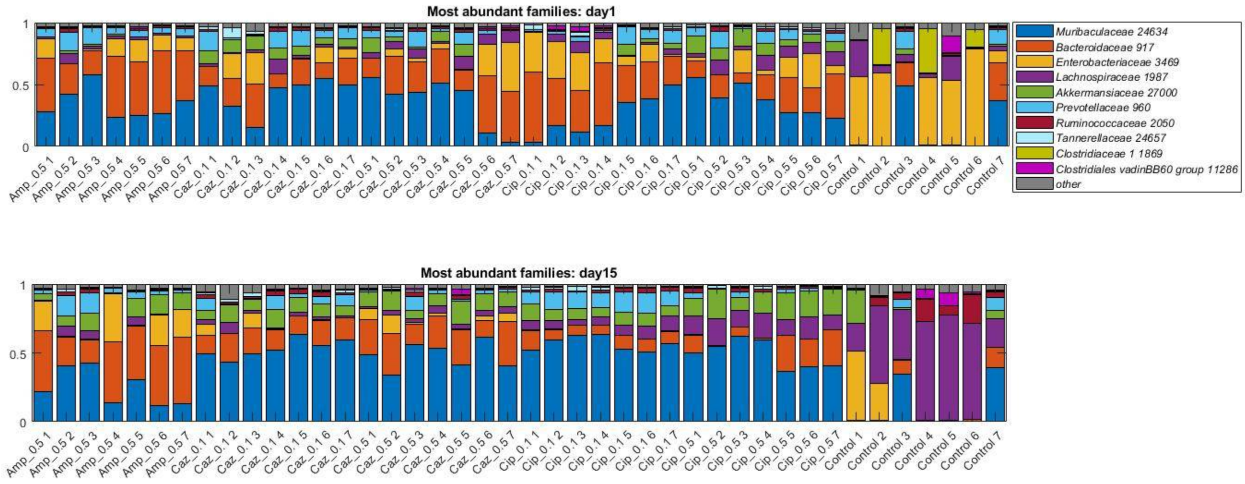

Figure S2. Relative abundancies at family level for each mice feaces sample on day 1st and day 15th that have at least 5% relative abundancy in at least one of the samples.

Supplementary Material S1

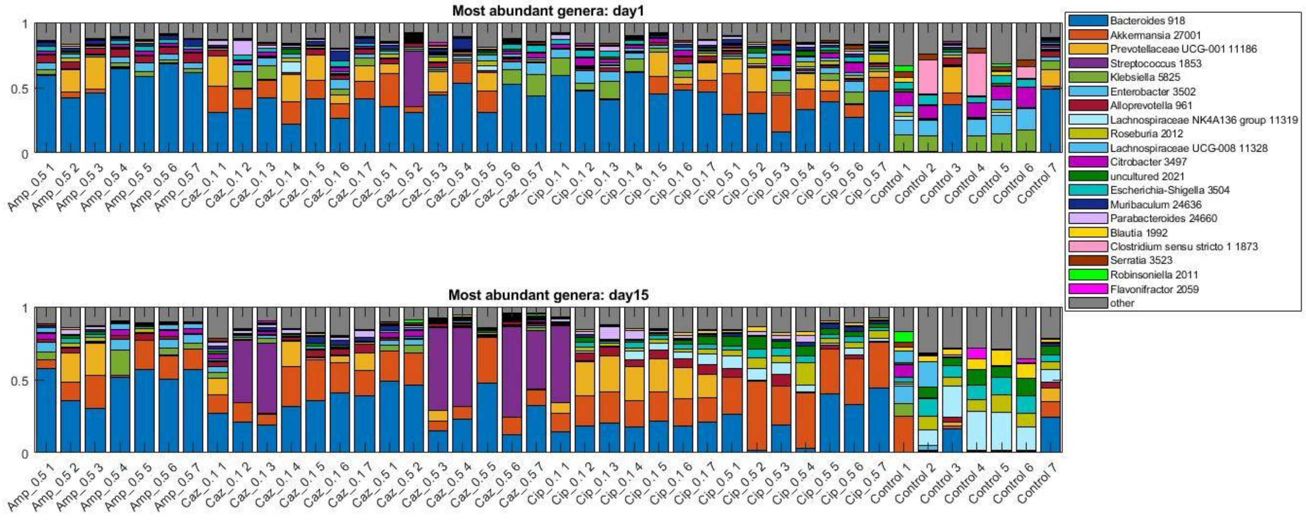

Figure S3. Relative abundancies at genus level for each mice feaces sample on day 1st and 15th day that have at least 5% relative abundancy in at least one of the samples.

Supplementary Material S1

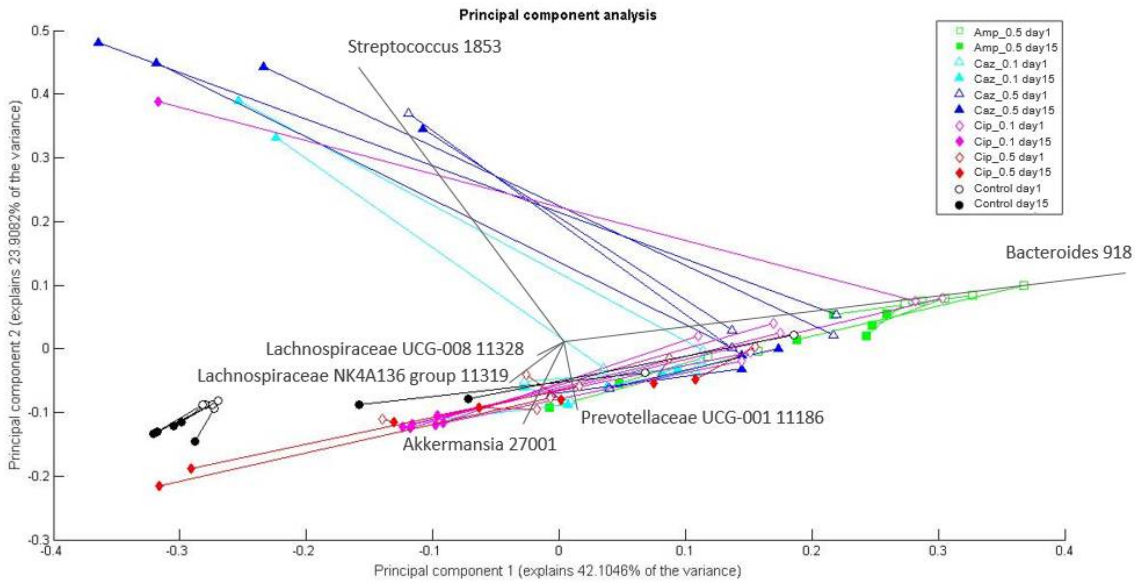

Figure S4. Principal component analysis of the different treatments and the changes in the composition of bacterial genera in the microbiome of each mice in time.

The % of variance explained by the component is shown on the axes; each marker represents one sample; colours correspond to the treatment (drug + dose) (Amp_0.5: green; Caz_0.1: cyan; Caz_0.5: blue; Cip_0.1: magenta; Cip_0.5: red; black: control); marker shapes correspond to the treatment (drug) administered. Legend: square: Amp; triangle: Caz; rhombus: Cip; circle: control; marker area refers to the sampling date (empty marker: day 1; full marker: day 15); lines connect the samples from the same source, so changes in a samples (in the PCA space) can be seen from the empty marker to the full one following the line between them. Arrows show the direction and importance of the original dimensions (the taxonomic categories) in the PCA space.

Supplementary Material S1

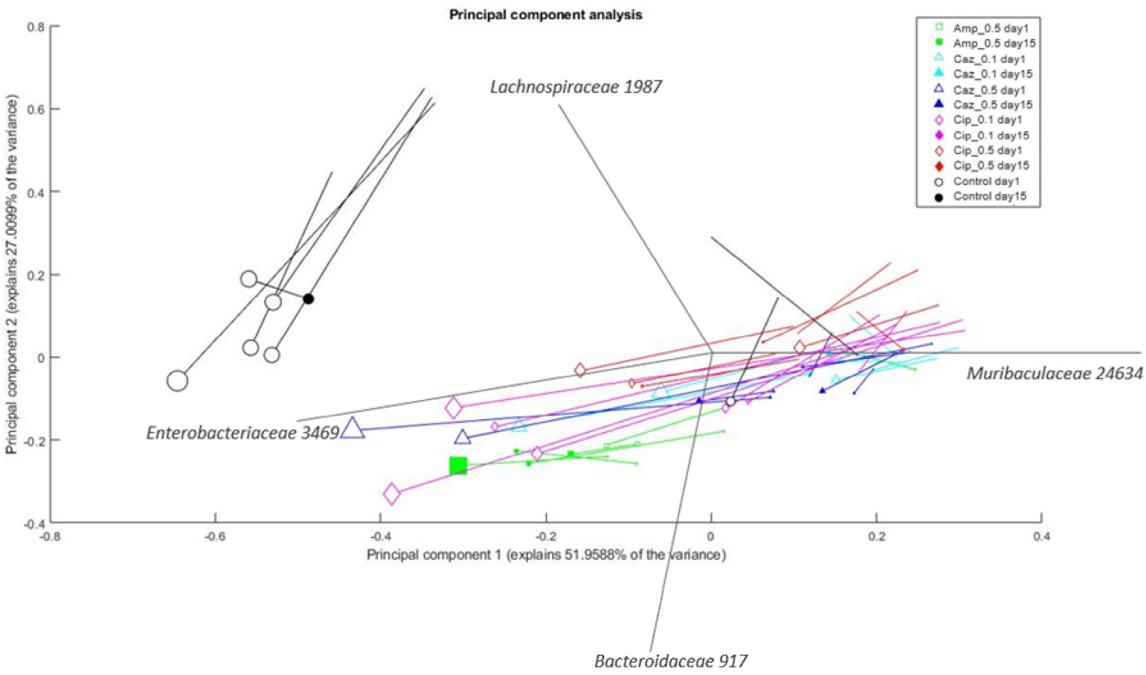

Figure S5. Principal component analysis of the different treatments and the changes in the composition of bacterial families in the microbiome of each mice in time.

The % of variance explained by the component is shown on the axes; each marker represents one sample; colours correspond to the treatment (drug + dose) (Amp_0.5: green; Caz_0.1: cyan; Caz_0.5: blue; Cip_0.1: magenta; Cip_0.5: red; black: control); marker shapes correspond to the treatment (drug) administered. Legend: square: Amp; triangle: Caz; rhombus: Cip; circle: control; marker area refers to the sampling date (empty marker: day 1; full marker: day 15); lines connect the samples from the same source, so changes in a samples (in the PCA space) can be seen from the empty marker to the full one following the line between them. Marker size correlates with the Klebsiella content (relative abundance) of the sample. Arrows show the direction and importance of the original dimensions (the taxonomic categories) in the PCA space.

Supplementary Material S1

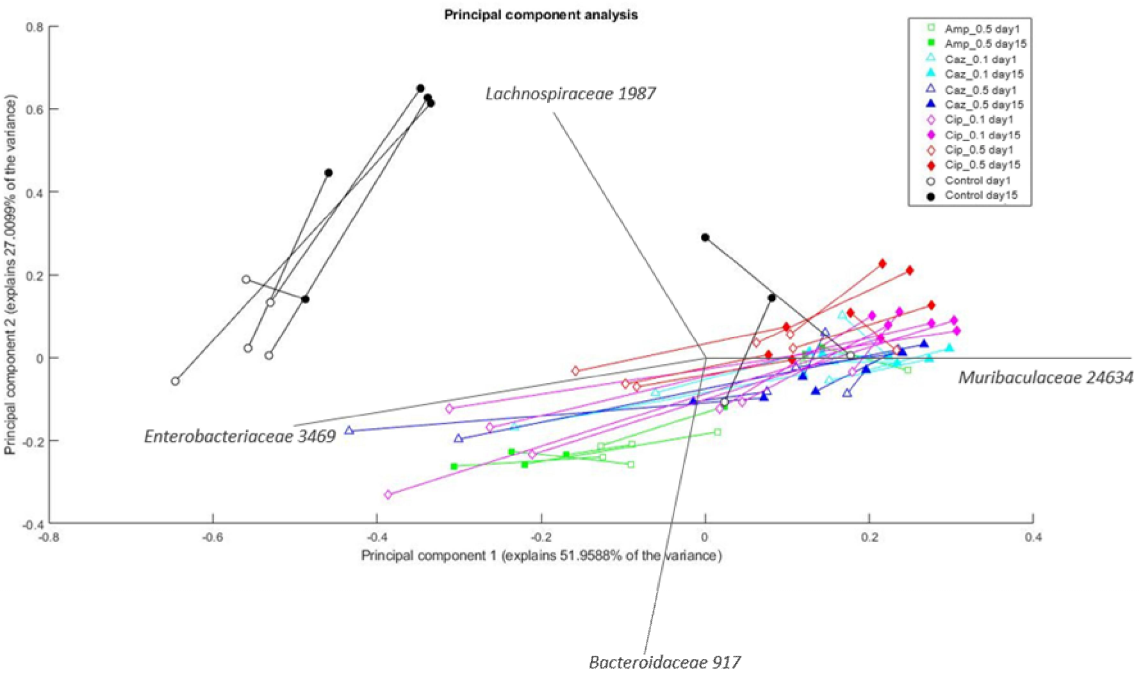

Figure S6. Principal component analysis of the different treatments and the changes in the composition of bacterial families in the microbiome of each mice in time.

The % of variance explained by the component is shown on the axes; each marker represents one sample; colours correspond to the treatment (drug + dose) (Amp_0.5: green; Caz_0.1: cyan; Caz_0.5: blue; Cip_0.1: magenta; Cip_0.5: red; black: control); marker shapes correspond to the treatment (drug) administered. Legend: square: Amp; triangle: Caz; rhombus: Cip; circle: control; marker area refers to the sampling date (empty marker: day 1; full marker: day 15); lines connect the samples from the same source, so changes in a samples (in the PCA space) can be seen from the empty marker to the full one following the line between them. Arrows show the direction and importance of the original dimensions (the taxonomic categories) in the PCA space.

Supplementary Material S1

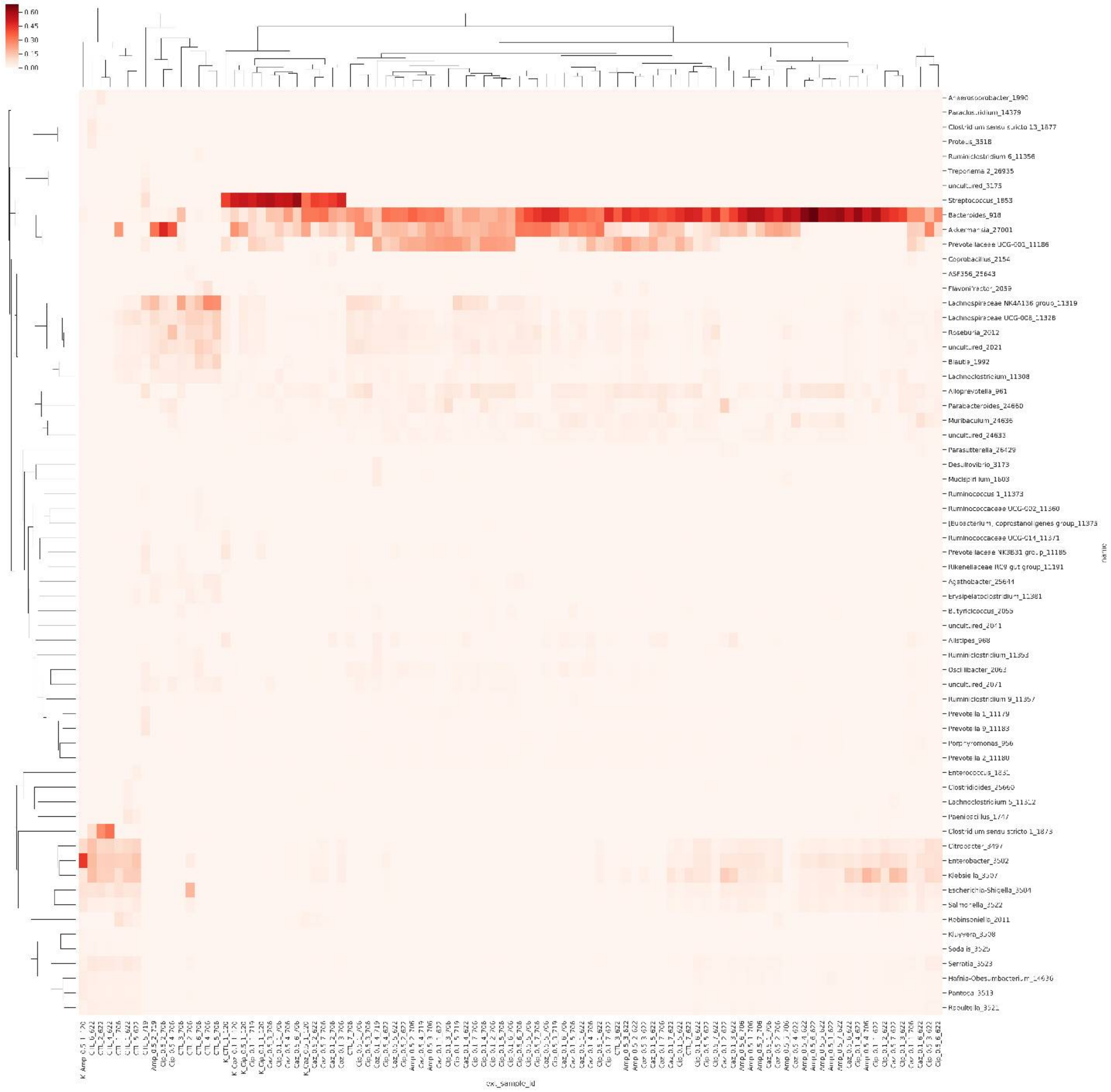

Figure S7. Correlation between Klebsiella and other genera. Proliferation rates of bacteria from the stool samples of each mouse addressed to different treatments at different timepoints.

The heatmap shows bacteria that were represented in the faeces at least in 1%. We could observe two types of bacterial groups, one group containing bacteria that changed their proliferation level at the same rate and another bacterial group in which that the trend of proliferation rate moved in opposite direction. The values at the x and y axes were clustered based on Bray-Curtis distances. Colors are corresponding to the rho values: deeper red colors represent stronger positive correlations.

Supplementary Material S1

Table S1. Pairwise Spearman correlations between Klebsiella and and other bacterial genera with |rho|≥0.4 and p-value<0.05.

Spearman rho	p-value	Bacteria pairwise
0,966501	3,07E-25	Citrobacter 3497 - Klebsiella 5825
0,911455	5,09E-17	Enterobacter 3502 - Klebsiella 5825
0,882772	1,06E-14	Raoultella 3521 - Klebsiella 5825
0,869518	7,97E-14	Pantoea 3513 - Klebsiella 5825
0,865884	1,33E-13	Sodalis 3525 - Klebsiella 5825
0,859146	3,33E-13	endosymbionts4 14649 - Klebsiella 5825
0,855837	5,13E-13	Halomonas 3616 - Klebsiella 5825
0,85113	9,32E-13	Erwinia 3503 - Klebsiella 5825
0,844322	2,13E-12	Vibrio 3783 - Klebsiella 5825
0,84245	2,66E-12	Escherichia-Shigella 3504 - Klebsiella 5825
0,825905	1,67E-11	endosymbionts8 14653 - Klebsiella 5825
0,80694	1,09E-10	Aeromonas 3318 - Klebsiella 5825
0,802622	1,63E-10	Kluyvera 3508 - Klebsiella 5825
0,789727	5,08E-10	Pectobacterium 3514 - Klebsiella 5825
0,783514	8,55E-10	Candidatus Rosenkranzia 3490 - Klebsiella 5825
0,781873	9,78E-10	Salmonella 3522 - Klebsiella 5825
0,778648	1,27E-09	Shimwellia 3524 - Klebsiella 5825
0,77616	1,55E-09	Serratia 3523 - Klebsiella 5825
0,734278	3,12E-08	Thorsellia 3527 - Klebsiella 5825
0,704602	1,91E-07	Kosakonia 14637 - Klebsiella 5825
0,684741	5,70E-07	Candidatus Regiella 3488 - Klebsiella 5825
0,682043	6,57E-07	Hafnia-Obesumbacterium 14636 - Klebsiella 5825
0,62676	8,97E-06	Colwellia 3379 - Klebsiella 5825
0,614831	1,48E-05	Robinsoniella 2011 - Klebsiella 5825
0,602941	2,38E-05	Dickeya 3500 - Klebsiella 5825
0,598834	2,80E-05	Massilia 26414 - Klebsiella 5825
0,593446	3,44E-05	Photorhabdus 3515 - Klebsiella 5825

Supplementary Material S1

0,589993	3,92E-05	Alcaligenes 26358 - Klebsiella 5825
0,523454	3,75E-04	Morganella 3512 - Klebsiella 5825
0,512841	5,15E-04	Pseudomonas 3723 - Klebsiella 5825
0,506733	6,16E-04	Pluralibacter 14641 - Klebsiella 5825
0,480262	1,29E-03	Photobacterium 3781 - Klebsiella 5825
0,47432	1,51E-03	Providencia 3519 - Klebsiella 5825
0,472871	1,56E-03	Shewanella 3400 - Klebsiella 5825
0,466446	1,85E-03	Arsenophonus 3470 - Klebsiella 5825
0,466446	1,85E-03	Candidatus Stammerula 3492 - Klebsiella 5825
0,464877	1,92E-03	Alteromonas 3339 - Klebsiella 5825
0,464093	1,96E-03	Cronobacter 3499 - Klebsiella 5825
0,41037	6,95E-03	uncultured 11548 - Klebsiella 5825
0,378276	1,35E-02	Candidatus Azobacteroides 24603 - Klebsiella 5825
0,369612	1,60E-02	Lonsdalea 3510 - Klebsiella 5825
0,363314	1,80E-02	Buchnera 3473 - Klebsiella 5825
0,362859	1,82E-02	Desulfovibrio 3173 - Klebsiella 5825
0,35384	2,15E-02	Proteus 3518 - Klebsiella 5825
0,336257	2,95E-02	Thiomicrorhabdus 26856 - Klebsiella 5825
0,329566	3,31E-02	Sphingobacterium 25032 - Klebsiella 5825
0,320504	3,85E-02	uncultured 26195 - Klebsiella 5825
0,308656	4,67E-02	uncultured 24558 - Klebsiella 5825
-0,307597	4,75E-02	Catonella 1994 - Klebsiella 5825
-0,30858	4,68E-02	Dysgonomonas 24605 - Klebsiella 5825
-0,314172	4,27E-02	Anaerocolumna 25646 - Klebsiella 5825
-0,3181	4,01E-02	Tannerella 24661 - Klebsiella 5825
-0,323411	3,67E-02	Rs-D38 termite group 974 - Klebsiella 5825
-0,326012	3,51E-02	Clostridium sensu stricto 1 1873 - Klebsiella 5825
-0,326118	3,51E-02	Harryflintia 25667 - Klebsiella 5825
-0,326859	3,46E-02	[Eubacterium] oxidoreducens group 11339 - Klebsiella 5825
-0,326867	3,46E-02	Herbinix 25653 - Klebsiella 5825

Supplementary Material S1

-0,326867	3,46E-02	Ruminiclostridium 6 11356 - Klebsiella 5825
-0,326867	3,46E-02	uncultured 1868 - Klebsiella 5825
-0,327643	3,42E-02	Saccharofermentans 2068 - Klebsiella 5825
-0,339075	2,80E-02	Parabacteroides 24660 - Klebsiella 5825
-0,340914	2,72E-02	Ruminiclostridium 5 11355 - Klebsiella 5825
-0,341342	2,69E-02	Macellibacteroides 24659 - Klebsiella 5825
-0,341825	2,67E-02	Johnsonella 2002 - Klebsiella 5825
-0,346125	2,47E-02	[Eubacterium] ruminantium group 11340 - Klebsiella 5825
-0,347707	2,41E-02	Howardella 2000 - Klebsiella 5825
-0,351553	2,24E-02	Porphyromonas 956 - Klebsiella 5825
-0,362838	1,82E-02	Ruminiclostridium 9 11357 - Klebsiella 5825
-0,367788	1,66E-02	Prevotella 963 - Klebsiella 5825
-0,369246	1,61E-02	Dorea 1997 - Klebsiella 5825
-0,375824	1,42E-02	Proteiniphilum 24608 - Klebsiella 5825
-0,376404	1,40E-02	uncultured 2071 - Klebsiella 5825
-0,379931	1,31E-02	[Eubacterium] hallii group 11338 - Klebsiella 5825
-0,380537	1,29E-02	Lachnospiraceae NC2004 group 11316 - Klebsiella 5825
-0,380818	1,28E-02	Tyzzerella 4 11336 - Klebsiella 5825
-0,386434	1,15E-02	Lachnospiraceae UCG-001 11321 - Klebsiella 5825
-0,388657	1,10E-02	Ruminococcaceae UCG-003 11361 - Klebsiella 5825
-0,389881	1,07E-02	Oscillospira 2064 - Klebsiella 5825
-0,390085	1,07E-02	Lachnospiraceae XPB1014 group 11331 - Klebsiella 5825
-0,394045	9,82E-03	[Eubacterium] ventriosum group 11341 - Klebsiella 5825
-0,398357	8,98E-03	Candidatus Vestibaculum 24658 - Klebsiella 5825
-0,398368	8,98E-03	Marvinbryantia 2005 - Klebsiella 5825
-0,405757	7,68E-03	Barnesiella 24598 - Klebsiella 5825
-0,406806	7,50E-03	Ruminococcaceae UCG-005 11363 - Klebsiella 5825
-0,409583	7,07E-03	Butyrivibrio 2 11300 - Klebsiella 5825

Supplementary Material S1

-0,416286	6,10E-03	Ruminococcaceae UCG-013 11370 - Klebsiella 5825
-0,420314	5,58E-03	A2 25642 - Klebsiella 5825
-0,42077	5,53E-03	GCA-900066225 25666 - Klebsiella 5825
-0,420878	5,51E-03	Ruminiclostridium 11353 - Klebsiella 5825
-0,421046	5,49E-03	Rikenellaceae RC9 gut group 11191 - Klebsiella 5825
-0,422415	5,32E-03	Prevotella 1 11179 - Klebsiella 5825
-0,429219	4,56E-03	Prevotella 7 11182 - Klebsiella 5825
-0,43209	4,27E-03	Ruminococcaceae UCG-002 11360 - Klebsiella 5825
-0,43528	3,96E-03	Negativibacillus 25668 - Klebsiella 5825
-0,439943	3,55E-03	ASF356 25643 - Klebsiella 5825
-0,443062	3,30E-03	Oscillibacter 2063 - Klebsiella 5825
-0,447773	2,94E-03	Lachnospiraceae AC2044 group 11313 - Klebsiella 5825
-0,449446	2,83E-03	[Eubacterium] xylanophilum group 14375 - Klebsiella 5825
-0,449831	2,80E-03	Prevotellaceae UCG-001 11186 - Klebsiella 5825
-0,450688	2,74E-03	Ruminococcaceae NK4A214 group 11358 - Klebsiella 5825
-0,451759	2,67E-03	Ruminococcus 2 11374 - Klebsiella 5825
-0,45226	2,64E-03	Lachnospira 2004 - Klebsiella 5825
-0,456031	2,40E-03	Hungatella 11306 - Klebsiella 5825
-0,464048	1,96E-03	Candidatus Soleaferrea 11350 - Klebsiella 5825
-0,471263	1,63E-03	Lachnospiraceae ND3007 group 11317 - Klebsiella 5825
-0,472514	1,58E-03	[Eubacterium] eligens group 14372 - Klebsiella 5825
-0,477444	1,39E-03	Blautia 1992 - Klebsiella 5825
-0,484692	1,14E-03	Ruminococcaceae UCG-010 11367 - Klebsiella 5825
-0,496187	8,32E-04	[Ruminococcus] gauvreauii group 11342 - Klebsiella 5825
-0,513659	5,03E-04	Ruminococcaceae UCG-014 11371 - Klebsiella 5825
-0,528107	3,25E-04	Acetitomaculum 1989 - Klebsiella 5825
-0,530126	3,05E-04	Lachnospiraceae UCG-006 11326 - Klebsiella 5825
-0,530396	3,03E-04	uncultured 964 - Klebsiella 5825

Supplementary Material S1

-0,534768	2,64E-04	Ruminococcus 1 11373 - Klebsiella 5825
-0,534943	2,62E-04	Lachnospiraceae UCG-008 11328 - Klebsiella 5825
-0,535559	2,57E-04	Faecalibacterium 2057 - Klebsiella 5825
-0,547195	1,77E-04	Acetatifactor 1988 - Klebsiella 5825
-0,550435	1,59E-04	Lachnoclostridium 10 11310 - Klebsiella 5825
-0,551872	1,51E-04	[Ruminococcus] torques group 14377 - Klebsiella 5825
-0,55868	1,20E-04	Odoribacter 24616 - Klebsiella 5825
-0,58843	4,16E-05	Fusicatenibacter 11305 - Klebsiella 5825
-0,59768	2,92E-05	[Eubacterium] coprostanoligenes group 11375 - Klebsiella 5825
-0,598362	2,85E-05	Oribacterium 2008 - Klebsiella 5825
-0,609958	1,80E-05	Lachnospiraceae NK3A20 group 11318 - Klebsiella 5825
-0,615631	1,43E-05	Agathobacter 25644 - Klebsiella 5825
-0,624699	9,79E-06	Tyzzerella 3 11335 - Klebsiella 5825
-0,631281	7,38E-06	uncultured 2021 - Klebsiella 5825
-0,675595	9,16E-07	Lachnoclostridium 11308 - Klebsiella 5825
-0,689624	4,39E-07	Lachnospiraceae UCG-004 11324 - Klebsiella 5825
-0,696975	2,94E-07	Anaerostipes 1991 - Klebsiella 5825
-0,698115	2,76E-07	Roseburia 2012 - Klebsiella 5825
-0,707483	1,62E-07	Lachnospiraceae NK4A136 group 11319 - Klebsiella 5825
